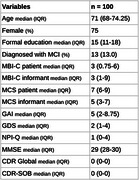# Assessment of the mild behavioral impairment checklist in individuals with subjective cognitive decline and mild cognitive impairment in the BRASCODE cohort

**DOI:** 10.1002/alz70857_106800

**Published:** 2025-12-26

**Authors:** Victória Tizeli Souza, Barbara Loeblein Uebel, Gabriela Raquel Paz Rivas, Simone Sieben da Mota, Bruno De Oliveira De Marchi, Guilherme Da Silva Carvalho, Haniel Bispo De Souza, Lucas Bastos Beltrami, Rhaná Carolina Santos, Sarah Vitoria Bristot Carnevalli, Ana Letícia Amorim de Albuquerque, Leonardo Martins de Paula, Manuella Edler Zandoná Giordani, Wyllians Vendramini Borelli, Giovanna Carello‐Collar, Marcia L. Chaves, Eduardo R. Zimmer, Raphael Machado Castilhos

**Affiliations:** ^1^ Federal University of Rio Grande do Sul, Porto Alegre, Rio Grande do Sul, Brazil; ^2^ Hospital de Clínicas de Porto Alegre, Porto Alegre, RS, Brazil; ^3^ Universidade Federal do Rio Grande do Sul, Porto Alegre, RS, Brazil; ^4^ Hospital de Clínicas de Porto Alegre, Porto Alegre, Rio Grande do Sul, Brazil; ^5^ Universidade do Vale do Rio dos Sinos, São Leopoldo, Rio Grande do Sul, Brazil; ^6^ Universidade Federal de Santa Catarina, Florianópolis, Santa Catarina, Brazil; ^7^ Centro de Memória, Hospital Moinhos de Vento, Porto Alegre, RS, Brazil; ^8^ Clinical Hospital of Porto Alegre, Porto Alegre, Rio Grande do Sul, Brazil; ^9^ Hospital Moinhos de Vento, Porto Alegre, Rio Grande do Sul, Brazil; ^10^ Brain Institute of Rio Grande do Sul (InsCer), PUCRS, Porto Alegre, Rio Grande do Sul, Brazil; ^11^ Hospital de Clinicas de Porto Alegre, Porto Alegre, RS, Brazil; ^12^ UFRGS, Porto Alegre, Brazil; ^13^ McGill University, Montreal, QC, Canada; ^14^ Brain Institute of Rio Grande do Sul ‐ Pontifícia Universidade Católica do Rio Grande do Sul, Porto Alegre, Rio Grande do Sul, Brazil; ^15^ Universidade Federal do Rio Grande do Sul, Porto Alegre, Rio Grande do Sul, Brazil

## Abstract

**Background:**

Mild Behavioral Impairment (MBI) is characterized by neuropsychiatric symptoms that may precede cognitive decline in the early stages of neurodegenerative diseases. In Brazil, the study of MBI in individuals with cognitive complaints remains limited. This study aims to evaluate the MBI‐Checklist (MBI‐C) in individuals with Subjective Cognitive Decline (SCD) and Mild Cognitive Impairment (MCI) in the Brazilian Subjective Cognitive Decline (BRASCODE) cohort in southern Brazil.

**Methods:**

Cognitively unimpaired adults >65 years old with cognitive complaints, but no severe clinical or neuropsychiatric illness, are enrolled in the BRASCODE cohort. This analysis includes data from participants who completed the 12‐month follow‐up. The applied scales included: Mild Behavioral Impairment Checklist (MBI‐C) and Memory Complaint Scale (MCS) (participant and informant); Neuropsychiatric Inventory–Questionnaire (NPI‐Q) (informant); Geriatric Depression Scale (GDS), Geriatric Anxiety Inventory (GAI), Mini‐Mental State Examination (MMSE) (participant); and Clinical Dementia Rating (CDR). We performed a correlation analysis between continuous variables. We considered a *p*‐value < 0.05 as statistically significant.

**Results:**

A total of 100 participants completed the 12‐month follow‐up, of whom 13 were diagnosed with MCI. Table 1 presents demographic and clinical characteristics. MBI‐C participant scores correlated positively with depressive (GDS, rho = 0.695, *p* < 0.0001) and anxiety symptoms (GAI, rho = 0.519, *p* < 0.0001). MBI‐C informant scores showed a strong correlation with NPI‐Q (rho = 0.743, *p* < 0.0001) and the informant's perception of cognitive decline (rho = 0.364, *p* =  0.0004). A significant correlation was also found between MBI‐C informant scores and CDR‐SOB (rho = 0.283, *p* =  0.007). Additionally, MBI‐C participant scores were negatively correlated with formal education (rho = ‐0.387, *p* =  0.009). No significant correlation was found between MBI‐C‐participant and MBI‐informant scores.

**Conclusion:**

The MBI‐C identified neuropsychiatric symptoms in individuals with SCD and MCI, showing a strong correlation with anxiety and depressive symptoms. Additionally, MBI‐C‐informant scores were significantly correlated with neuropsychiatric symptoms and cognitive impairment measures. These findings bring attention to the relevance of MBI‐C in the behavioral assessment of this population. Research with larger samples is needed to better understand the associations between MBI and cognitive decline in individuals with SCD and MCI.